# ‘Experiencing one thing and saying another’–Ecological Momentary Assessment (EMA) of nursing students’ competence and challenge during clinical placements compared with retrospective interviews

**DOI:** 10.1371/journal.pone.0302866

**Published:** 2024-05-22

**Authors:** Klas Karlgren, Mikael Andersson Franko, David Kilström

**Affiliations:** 1 Department Learning, Informatics, Management and Ethics, Karolinska Institutet, Stockholm, Sweden; 2 Department of Research, Education, Development and Innovation, Södersjukhuset, Stockholm, Sweden; 3 Faculty of Health and Social Sciences, Western Norway University of Applied Sciences, Bergen, Norway; 4 Department of Clinical Science and Education, Karolinska Institutet, Södersjukhuset, Södersjukhuset, Stockholm, Sweden; Örebro University Faculty of Medicine and Health: Orebro universitet Fakulteten for medicin och halsa, SWEDEN

## Abstract

Clinical placements are essential to nursing education and understanding students’ challenges in the clinical context is important for educators. Nevertheless, few studies have investigated students’ experiences in the clinical context itself but rely on methods which ask participants to generalize their clinical experiences retrospectively. **Objectives**: This study aimed to explore nursing students’ experiences of clinical activities during and after clinical placements with a focus on feelings of competence and challenge. A particular interest was on comparing momentary assessments in the clinical context with retrospective interview data. **Methods**: Smartphones were used for ecological momentary assessment of students’ experiences of clinical activities during five-week placements at 21 nursing homes. Both quantitative and qualitative data were collected. Interviews were conducted after the placements. **Results**: 575 responses were obtained showing final-year nursing students rated their competence significantly higher and challenge significantly lower than first-year students. An analysis of the quantitative data using the four-channel flow model showed that first-year students’ activities were to a significantly higher extent associated with flow and anxiety, compared to those of final-year students. Conversely, the final-year students’ activities were to a significantly higher extent associated with boredom than those of first-year students. The analysis of the students’ reflections resulted in five themes: *Specific activities are challenging*, *Lack of challenge*, *Learning and developing*, *Reflects critically on competence*, and *Arrangement of the clinical placement*. **Discussion**: Final-year nursing students were significantly less challenged during the placements compared to first-year students. There was overlap in how students described their clinical experiences during and after placements, but there were also striking differences. The first-year students were overwhelmed by the challenges during the placements but viewed these experiences as valuable learning experiences afterwards. The final-year students also described several challenging clinical activities during the placements but none of these challenges were brought up afterwards in the interviews and instead a lack of challenge was emphasized. Momentary assessment thus enabled capturing data about clinical activities which may be overlooked by retrospective methods.

## Introduction

Clinical placements are essential to nursing education [1]. However, the majority of existing studies rely on retrospective generalizations based on what students recall of their clinical experiences. Few studies have investigated students’ experiences and challenges in the clinical context itself. The latter may be necessary for ensuring that activities at placements are considered meaningful, that learning follows a reasonable progression, and adequate supervision is provided.

Preparing students for the transition to work life practices is a growing concern in education in general, in particular when the requirements on work life competences are changing faster than before [2]. This is especially clear in healthcare and nursing where the educational system is criticized for not sufficiently preparing nurses to fully participate in patient care while the practice settings are criticized for having unrealistic expectations of new graduates [3]. Research has suggested that nursing students may be so overwhelmed in the clinical setting that safety is compromised [4]. Killam & Heerschap have stressed the need for better understanding of student challenges within the clinical setting as these may hinder learning [5] and because negative clinical experiences have been shown to influence student confidence contributing to nursing students leaving the profession. Nursing homes may, for instance, be surrounded by (possibly groundless) beliefs of being undemanding, depressing, or not adding to skills and competencies that students need in their future practice [6–8].

In most cases, studies about clinical placements rely on survey instruments, interviews, or focus group interviews asking retrospective questions about previous experiences. Such methods are comparatively easy to administer but have been criticized for relying on retrospective generalizations which are prone to various forms of biases and distortions. Respondents are asked to select, recall and summarize events after the fact despite the well-known risks of forgetting, overlooking, embellishing, or exaggerating their responses as well as being influenced by social desirability bias. There is an advantage of examining behaviors in their natural settings [9]. During the last couple of decades, ecological momentary assessment methods have become more established (other names are contextual activity sampling, experience sampling methods, daily diary methods, ambulatory assessment). Ecological momentary assessment allows data about behaviors, thoughts, and feelings in daily life to be collected in situ on multiple occasions over time using ordinary mobile phones [10–12]. This approach eliminates retrospection bias, provides data representing behaviors across multiple occasions and ensures ecological validity by examining data in their natural context [9]. For instance, a study using a contextual activity sampling approach showed how students’ learning experiences and academic emotions were connected to specific activities and how they developed over time on an interprofessional training ward [13]. Interestingly, the research approach was able to identify negative experiences and problems in the clinical context which were not captured by retrospective questionnaires or interviews [11].

Finding a reasonable balance between challenges and skills is a concern for every educator. The terms competence, competency, skill, capability, capacity, and ability are often used interchangeably or with specific meanings cited in the literature [14]. Competence is sometimes defined as an overall concept that refers to the ability to manage in a specific context and which may be formed by a set of capacities and skills [14]. In this study, we take competence to refer to the knowledge and skills enabling a person to act and work intentionally and effectively both individually and together with others in the clinical context. A socio-cultural perspective places emphasis on participation in practices, thus competence development can be understood as the ability to take part in various clinical practices in progressively more expert ways. Flow theory has been intimately connected to experience sampling/ecological momentary assessment research where flow experiences (or optimal experiences) are characterized by intense and focused concentration on the present moment, having a sense of personal control or agency over the activity, and experiencing the activity as intrinsically rewarding [15]. The notion of flow has been important in educational research concerned with fostering positive learning experiences. Individuals are thought to operate at full capacity when in such a flow state. A condition for such a state is when perceived challenges stretch but do not overmatch existing skills making such experiences fragile: if challenges exceed skills one first becomes vigilant and then anxious and, conversely, if skills exceed challenges, one first relaxes and eventually becomes bored. The notion of flow therefore has important implications for the development of learning [16], e.g., when individuals develop the skills necessary to perform a certain activity, they also begin to master the challenges inherent in the activity and eventually the level of challenge drops. As skills are acquired, new challenges have to be identified to maintain a balance between challenges and skills. For educators to be able to support such a balance, research is needed investigating students’ experiences in the clinical context or there is a risk that learners’ challenges are not addressed adequately and that optimal conditions are not created for rewarding learning experiences. The aim of this study was to explore nursing students’ experiences of clinical activities during and after clinical placements with a focus on feelings of competence and challenge. A particular interest was on comparing momentary assessments in the clinical context with retrospective interview data.

## Material and methods

Our main research questions were: 1. How do nursing students rate, and reflect on, competence and challenge regarding clinical activities during their placements? 2. How are students’ experiences of clinical activities mapped into the four elements of the flow model? 3. How do nursing students describe their experiences at the clinical placements during and after the placements?

### Design of the study

This study collected data in the context of the clinical placement and retrospective interview data. Ecological momentary assessment (EMA) was chosen to gather students’ experiences over time using a smartphone app (see the next section for details). The app included queries concerning students’ experiences of challenge and competence in relation to clinical activities. Both qualitative and quantitative data were collected concurrently from the same participants to enhance understanding. Semi-structured follow-up interviews were conducted after the clinical placements.

### Participants and context of study

The study was conducted from 2018–2020 at 21 nursing homes. The participants were enrolled in a three-year nursing program in Stockholm, Sweden which includes mandatory, five-week clinical placements within elderly care during the second and sixth semesters. The program is arranged by Ersta-Sköndal-Bräcke University College which is the oldest nursing school in Sweden and organizes clinical placements in a large number of nursing homes. A convenience sampling strategy was used and all 44 first-year and all 44 final-year students were invited to participate. No students were excluded. Participants were offered the possibility to borrow smartphones, but all students had their own. Participants were recruited by first asking the students’ teachers to inform them about the study and then by an oral presentation made by two of the researchers (DK and KK) in the students’ classrooms. Additionally, written information was also provided.

The sample size was decided based on the goal of obtaining variation among experiences of clinical activities with an estimation—based on our previous research studies [11,13] concluding 400 responses were needed. We expected 20 participants to be appropriate assuming they delivered 20 responses each. Twenty-six students participated with the most common reason for non-participation being fear that participation would add stress during the clinical placements. All participating students were contacted via e-mail and telephone during the clinical placements to invite them to an interview after the placement. If the student was willing, an interview time was arranged. Fourteen students agreed to be interviewed with the most common reason for non-participation cited as a vacation period following the placement.

### Data collection

The students participated using the Expimetrics app (today renamed ExpiWell). The study focused on five daily queries during the second and fifth weeks of the clinical placements. In addition, spontaneous responses were also encouraged at any time during the clinical placement. A pre-placement questionnaire informed the participants about the study and collected information about the participants’ age, sex, and their nursing home assignment.

The daily queries asked which clinical activity the student was involved in at the time of responding, followed by rating scale questions asking about experienced levels of challenge and competence, and lastly, a free-text question (Table 1). The questions were previously developed for studying students’ learning experiences in clinical environments [11,13]. The participants were informed that the focus was on professional activities, i.e., relating to nursing, caring, and medicine, and that they should exclude activities which were not work-related (e.g., lunch breaks, private phone-calls etc.).

**Table 1:** Items in the daily queries (translated into English).

Nr	Item
**1**.	Which clinical activity are you involved in now? (choice between predefined options or free text input)
**2**.	How challenging do you experience this activity? (rating scale question)
**3**.	How competent do you feel when performing this activity? (rating scale question)
**4**.	Please tell us more. We are especially curious about your thoughts about learning, feedback, scientific evidence and participation (free text item)

To respond to questions 2–3, participants used a visual scale ranging from 0–100 with the anchor points “Not at all challenging” to “Very challenging” (item 2) and from “Not at all competent” to “Very competent” (item 3). The final question asked for comments in free text about their experiences. To support quick responses, the participants were able to select between predefined activities (*Working with patient record*, *Managing meal*, *Blood test*, *Showering/washing patient*, *Health maintenance*, *Administering medicine*, *Preparing medicines*, *Handling catheterization*, *Mobilizing patient*, *Help with personal hygiene*, *Toileting assistance*, *Injection*, *Inserting or managing urinary catheter*, *Interacting with healthcare personnel*, *Leading/planning/organizing care*, *Eating assistance*, *Blood or urine sampling*, *Dressing*, *Round*, *Rehabilitation*, *Dialogue*, *Dialogue with patient/relative*, *Cleaning and making bed*, *Wound dressing*, *Inserting urinary catheter*, *Brushing teeth*, *Giving a bed bath*, *Teaching*, *Following-up drug effects*, *Taking vital signs*) with free-text input also an option.

The daily queries were planned for certain times of the day, Monday-Friday at 8:30AM–11AM, 11AM–1:30PM, 1:30PM–4PM, 4PM–6:30PM, and 6:30PM–9PM. Reminders in the form of text and email messages were sent at the beginning of the second and fifth weeks of clinical practice and at the end of the placement. Reminders were also sent to participants who had not been active. When participants began responding, messages were sent encouraging them to continue.

Semi-structured, individual, face to face interviews were organized after the clinical placements conducted by DK and KK at the students’ university college, at the hospital where their next practice period took place, or in one case over telephone. All interviews were audio-recorded and transcribed verbatim and covered the themes presented in Table 2. Again, the participants were informed that the interest was in professional activities, in a broad sense, excluding non-work-related activities.

**Table 2:** Interview themes (translated into English).

Nr	Theme
**1**.	**Clinical activities**: How would you now, in retrospect, describe your experiences of your five-week placement (What did you learn? What was difficult? Which clinical activities have contributed the most to your learning?
**2**.	**Challenges**: How challenging have you experienced the clinical placement? What has been most challenging and why?
**3**.	**Competence**: How competent have you felt during the placement? When or in which situations have you felt most competent? When or in which situations have you felt least competent?
**4**.	A dialogue about participation in the study.

### Data analysis

Time trends of numerical data were analyzed with mixed linear models with week number and clinical activity as fixed effects and participant as random effect to adjust for individual variation. Differences between categories were tested using the chi-square test. Students’ responses about clinical activities were interpreted in terms of the balance of challenge and competence following the four-channel model generated by flow theory [17]. According to the model, the relationship between competence and challenge determines which “channel” or psychological state is experienced when involved in an activity. *Flow* is defined as a balance between high competence and high challenge during which the person stretches his or her capabilities with the likelihood of learning new skills and increasing self-esteem. This optimal experience is also associated with feeling more active, alert, concentrated, and creative. If, instead, the challenge is high and competence is low, the person will become overwhelmed by *anxiety*. And if challenge is low but competence is high, small efforts are needed to perform a task and the person will feel *bored*. Finally, if both the levels of challenge and competence are low, the person will experience *apathy*. Students’ responses were thus categorized into the model’s four categories as follows.

High assessments of challenge and competence were defined as flow.

High assessments (>50) of challenge and low assessments (<50) of competence were defined as anxiety.

Low assessments (<50) of challenge and high assessments (>50) of competence were defined as boredom.

Low assessments (<50) of both challenge and competence were defined as apathy.

In case of a response of exactly 50, every second response was interpreted as low and high, respectively. This was needed as the response scale (0–100) in the app had an uneven number of response options with a midpoint value. Analyses were performed using R (v3.6.0, 2021, Vienna, Austria). *P*-values of <0.05 were considered statistically significant.

A conventional, inductive content analysis was performed to analyze the free text responses and the interviews. The analysis focused on participants’ reflections about challenge and competence during their clinical placements and these were analyzed in their manifest form, i.e., the participants’ expressions and in their latent form, i.e., the underlying meaning. The transcripts were first read carefully to get a sense of the whole. The analysis was an iterative process identifying the participants’ descriptions of experiences and thoughts about competence and challenge. Meaning units were extracted, coded, grouped into categories and finally interpreted as themes representing the latent form of the findings. An example of the process is illustrated in Table 3. DK and KK first worked individually and then collaboratively in several meetings. The separate analyses were compared, and any differences were discussed until agreement was reached. Google Sheets was used as a collaboration tool and several coding trees were developed in Keynote.

**Table 3:** An example illustrating the content analysis process.

Reflections during clinical placements	Condensed meaning unit	Category	Theme
“Difficult to give subcutaneous injections to somebody without any body fat at all. I find it scary to give subcutaneous injections to someone who is extremely thin.”	Difficult to give subcutaneous injections	Clinical procedures	Specific activities are challenging
“Difficult conversation with a relative who expressed painful feelings about an incident with a parent. Hard to know what to say.”	Difficult conversation about relative’s feelings	Interaction with relatives	Specific activities are challenging

### Validity and reliability/Rigor

Credibility was ensured by continuously reflecting on and discussing the research focus and methods. Researcher triangulation was applied to reflect on data from different perspectives. The authors have experience of qualitative, quantitative, and mixed-methods studies. DK is a registered nurse and until recently a lecturer at a nursing school providing insight into the nursing students’ context. KK is a researcher in medical education with experience of several studies about students’ experiences of clinical placements. MAF is a biostatistician. This research project is based on the theoretical conviction that learning is not only an individual process but founded in social practices and physical contexts [18] and that learning often is a collaborative endeavor mediated by artifacts and technology [19]. Because of the fundamentally situated nature of human thinking we have chosen a research approach which builds on sampling of data in context, over time. Dependability has been ensured by descriptions of methodological choices and by continuously tracking and reporting discussions and decisions as above.

### Ethics approval and consent to participate

Ethical approval was sought from The Ethical Review Board in Stockholm which determined according to the Swedish Ethical Review Act that the study was exempt as this research did not entail handling of sensitive personal data (diary nr 2018/2252-31/5). Prior to the study, participants were informed orally, and in writing, that participation was voluntary—they could discontinue participation at any time without giving a reason, and filling in and submitting the queries was considered as giving consent. The same information was given in the app before the participants could respond to the questionnaires. All interview participants gave written consent. All methods were carried out in accordance with relevant guidelines and regulations.

## Results/Findings

In total, 575 daily queries were filled in by 26 students at 21 nursing homes, see Table 4.

**Table 4:** Participants and the number of responses during the clinical placements and interviews afterwards. In some cases, first- and final-year students were at the same nursing home which explains why the total number is 21 and not 23.

Nursing homes	Students and semester	Gender	Age	Daily queries	Interviews
9	10; first-year	9 female, 1 male	23–46	339	8
14	16; final-year	15 female, 1 male	22–40	236	6

**Fig 1:**
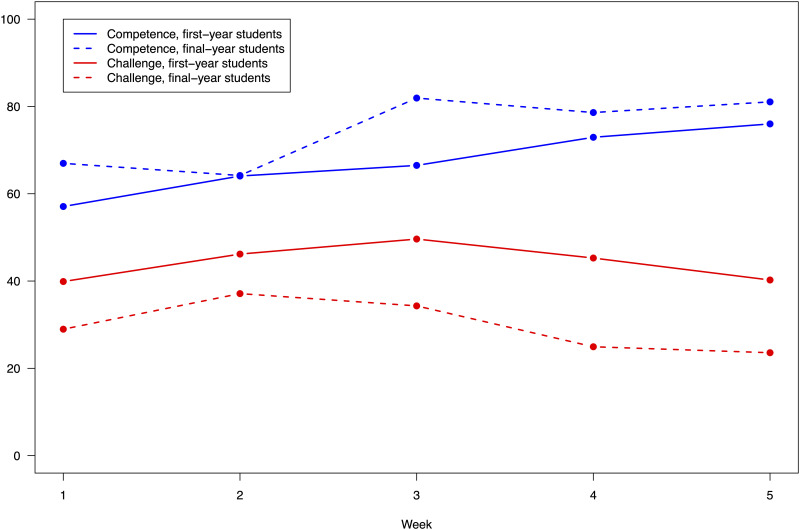
Competence and challenge over time as rated by first- and final-year students.

Both first- and final-year students rated their levels of competence relatively high, see Fig 1. The weekly means during the five weeks ranged between 57 and 76 out of 100 among the first-year students and between 64 and 82 among final-year students. The final-year students rated their levels of competence significantly higher than first-year students (*p* < 0.001). In contrast, the first-year students rated challenge significantly higher than the final-year students (*p* < 0.001). The first-year students’ mean ratings ranged between 40 and 50 while the final-year students’ ratings ranged between 24 and 37. Descriptive data are available as (S1–S3 Tables).

An analysis of the students’ assessments over time showed that both first- and final-year students’ assessments of competence increased significantly over time (*p* < 0.001 and *p* = 0.001, respectively). The rated levels of challenge reported by the students were rather stable over time although the final-year students lowered their assessments of challenge over time at the placements (*p* < 0.05).

**Fig 2:**
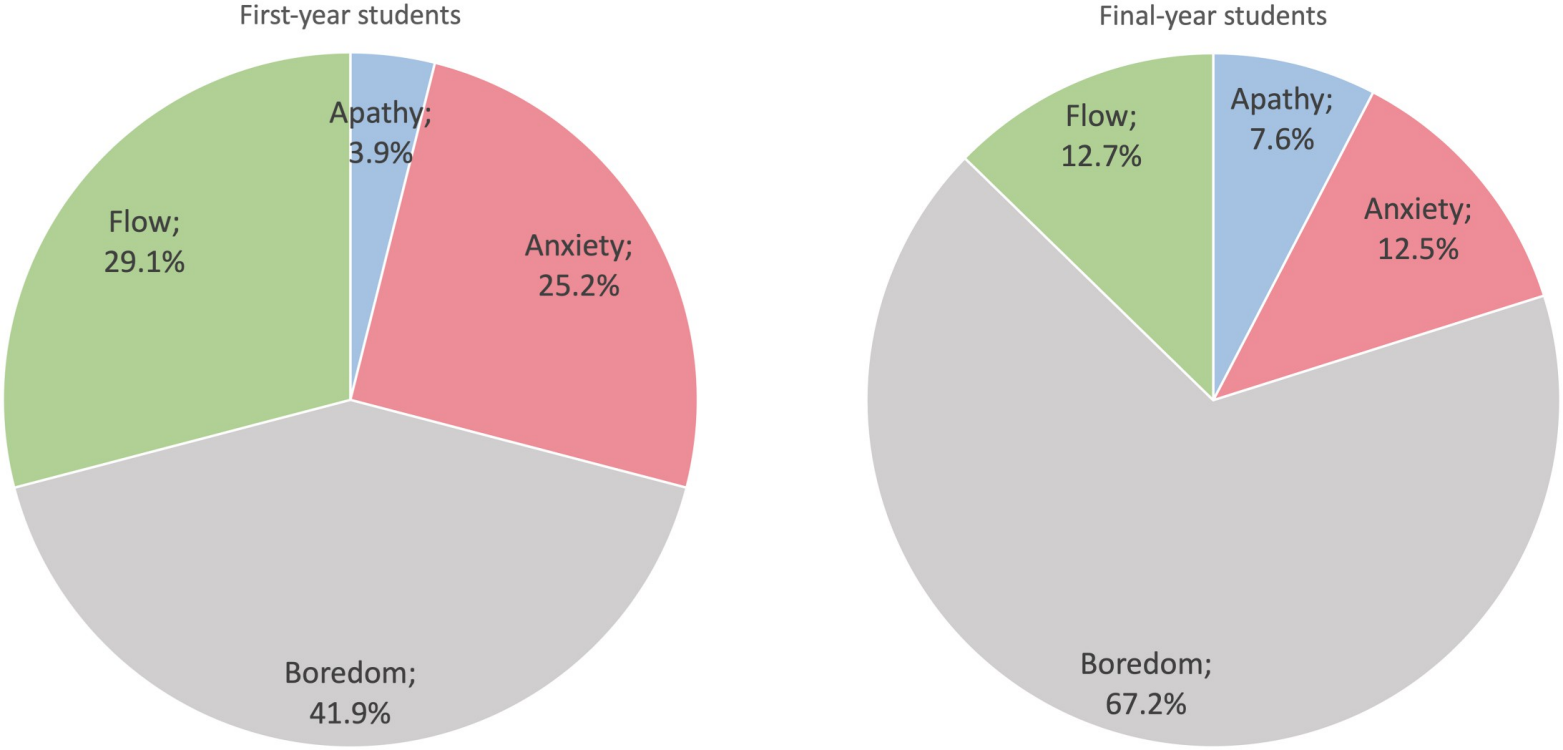
The distribution of students’ responses reported during the clinical placements categorized into the four elements of the flow model.

The students’ responses were analyzed using the four categories of the flow model. A chi-square test of the hypothesis that the students’ responses would be evenly distributed into the four quadrants (the flow categories) was rejected (*p* < 0.001). The focus of this analysis is on the students’ responses without distinguishing between clinical activities. As can be seen in Fig 2, the experiences of the first-year students were quite evenly distributed into the categories flow, boredom and anxiety but only a small fraction were categorized as belonging to apathy. In contrast, 74.8% of the reports from the final-year students were categorized as belonging to boredom and apathy. More than two thirds belonged to boredom. Considerably fewer responses among the final-year students belonged to flow and anxiety than among the first-year students. A chi-square test of the differences between first-year compared to final-year students’ reports showed significant differences in all categories except apathy: anxiety (*p* < 0.001), flow (*p* < 0.001), boredom (*p* < 0.001), and apathy (*p* = 0.083).

**Fig 3:**
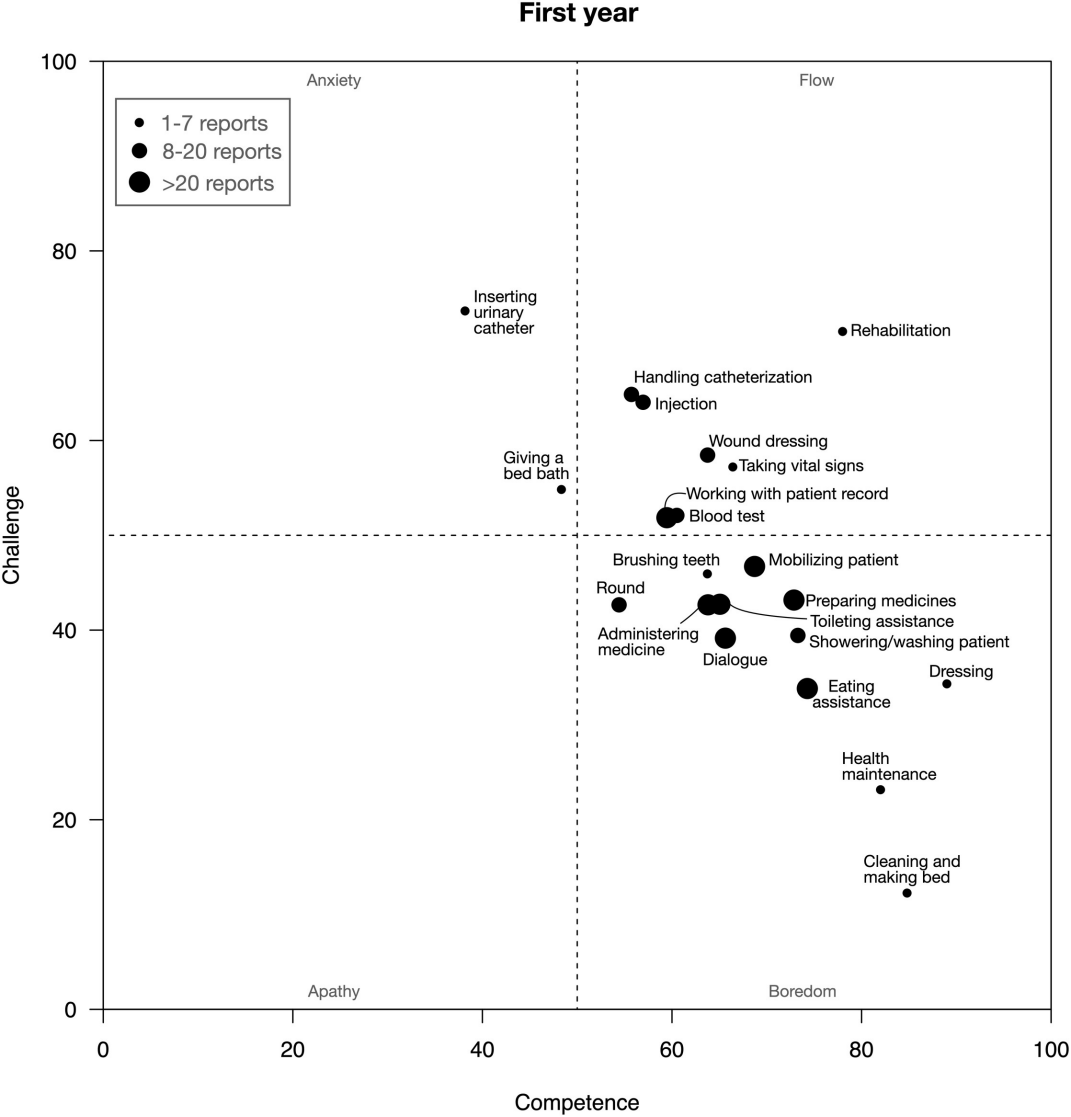
The graph shows how different activities are plotted into the four categories of the flow model, based on the first-year students’ assessments of challenge and competence.

**Fig 4:**
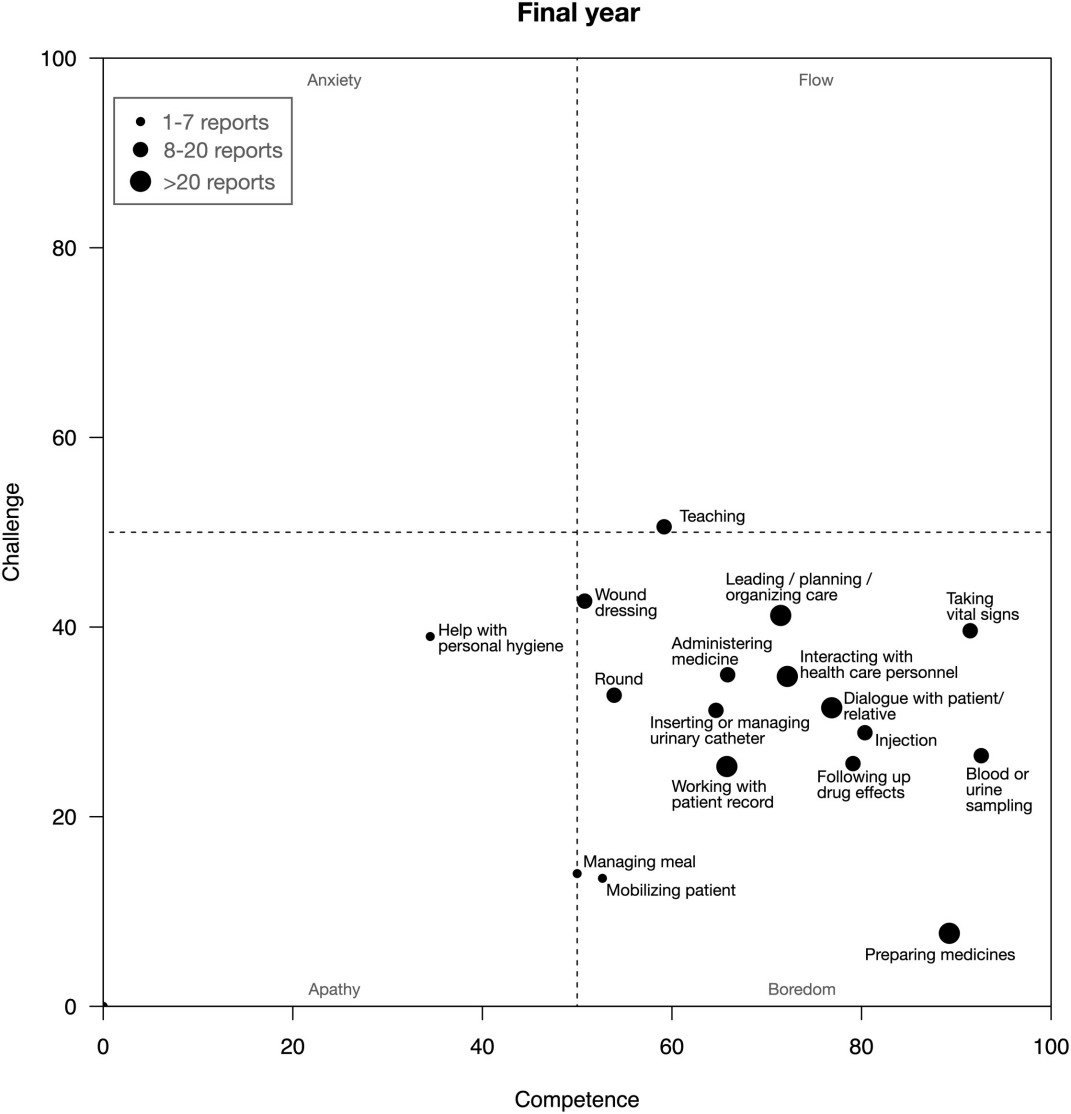
The graph shows how the different activities are plotted into the four categories of the flow model, based on the final-year students’ assessments of challenge and competence.

Figs 3 and 4 show how responses regarding different clinical activities were assessed on an average and plotted into the four different categories of the flow model. The graphs show that first-year students on an average assessed most activities as associated with flow or boredom while the final-year students associated most activities with boredom.

### Students’ reflections during and after the clinical placements

The free text responses made during the clinical placements and in the subsequent interviews showed that both first- and final-year students made frequent and elaborate reflections about experiences of challenge and competence. The analysis of the students’ reflections generated five themes, each with a number of categories (Table 5). These are presented below together with exemplifying quotes. Fuller excerpts from the free text responses and retrospective interviews are available as (S1 and S2 Texts).

**Table 5:** Students’ reflections in the free text comments during the clinical placements and in interviews after the placements presented as themes and underlying categories.

Theme	Category	Reflections *during* clinical practice		Reflections *afterwards* in interviews	
		First year	Final year	First year	Final year
**Specific activities are challenging**	Clinical procedures	✔	✔		
	Documentation	✔	✔		
	Interaction with the patient	✔	✔		
	Interaction with relatives		✔		
	Learning the equipment	✔			
**Lack of challenge**	Tasks are repetitive or under-stimulating		✔		✔
	Lack of structure and requirements				✔
	Tasks are easy but important	✔		✔	
**Learning and developing**	Confidence through independence and responsibility	✔	✔		✔
	Ability based on experience and training	✔	✔	✔	
	Observing others and being supervised	✔			
	Learning through leading, supporting, and supervising others		✔		
**Reflects critically on competence**	Questions the competence & behaviors of others	✔	✔	✔	
	Concerns about their own competence			✔	
	Appreciation of knowledge			✔	✔
**Arrangement of the clinical placement**	Questions activities and supervision		✔	✔	✔
	Balancing roles			✔	
	Constructive supervision			✔	

### Specific activities are challenging

The students reflected on numerous specific activities that were considered challenging during the placements. Specific *clinical procedures* were experienced as challenging with first-year students describing, e.g., taking blood samples, administering insulin, taking measurements, redressing wounds, and changing catheters. The final-year students also brought up several clinical procedures which they had experienced as challenging and which made them uncertain including giving subcutaneous injections and taking blood samples, catheterization, understanding medication lists and treating pain in the best way (“*Difficult to give subcutaneous injections to somebody without any body fat at all*. *I find it scary*…”).

Both student groups described *documentation* as challenging and interacting with the patient record systems as difficult. The first-year students brought up that learning and understanding the professional language, terminology and abbreviations was challenging (“…*Interesting but many difficult words and abbreviations*”). Final-year students noted challenges related to documenting (writing) the patient records without predefined terms to select from.

Both student groups identified *interaction with the patient* as challenging in many ways. The first-year students mentioned remembering to explain to the patient what they were doing, reaching agreements with patients about how to perform care (in cases when patients objected to what nursing students first had planned), responding to patients who are dissatisfied or refuse medicine, and discussing sensitive topics with patients suffering from anxiety (“*Dialogue about loneliness and death*, *difficult to know what to answer*”). Final-year students similarly reflected on challenges related to reaching agreements about medication, ethical aspects of caring patients who are unable to voice their opinions, feeling that they violate patients because they wear gloves when helping them with eating and moving patients from wheelchairs to beds etc.

The final-year students in addition commented on challenges regarding *interaction with relatives* of patients related to handling sensitive issues brought up by the relatives and explaining a terminal illness of a family member. (“…*Difficult*, *when relatives ask why their mother is dying*…”).

The first-year students brought up challenges related to *learning the equipment* such as understanding how to use wheelchairs, walking frames, rollators and air mattresses and knowing how to work in an ergonomically correct manner.

No mentions of any specific clinical activities that were challenging were made afterwards, in the retrospective interviews.

### Lack of challenge

Lack of challenge was a second distinct theme. The final-year students often commented that *tasks were repetitive or under-stimulating*. Both during and after the placements, tasks were described as too easy and even boring, in part because they already have extensive experience of the tasks and because they felt that they were not permitted to work independently enough, e.g., lacking the right to log in to read patient records and documenting on their own (“*I am feeling very under-stimulated at this nursing home*…”). Afterwards they would similarly point out that they had had to do the same kinds of things over and over again and that they had felt that they were wasting time not doing anything meaningful at all. Certain tasks, such as administering medication and taking vitals, were described as repetitive and not contributing to their learning. They expressed that time could have been used better and other, more challenging tasks (such as preparing intravenous treatments) would have contributed more to their learning (*“Every day*, *I felt*: *What am I doing here*? *What is this good for*? *Why have they chosen this*? *Many times we did not learn anything new and I just twiddled my thumbs”*).

After the placements the final-year students also expressed a *lack of structure and requirements*. They could experience the placements as unorderly and that they felt a need to create a structure themselves as they did not get help with planning days from supervisors. Supervisors did not seem to be interested in having students there and did not supervise, teach or focus on students’ learning as supervisors should. They were told that they were now nurses and expected to work as such on their own. They nevertheless did not feel that they had become colleagues but were rather considered to be a “*a tail or appendage*”.

In contrast, the first-year students described *tasks as easy but important*. These activities were described in positive ways, as undemanding but nevertheless important (“…*not especially challenging but an important task in the profession*”). In the interviews afterwards, these students said that they first had thought that the practice was unnecessary as they already had experience from health care and that it would just be repetition. But they also described how they eventually began thinking that the practice was in fact rewarding (“… *actually it was always rewarding if you think about it*, *but not in the beginning*”).

### Learning and developing

The third theme was learning and developing. One area of reflection concerned learning and growing *confidence through independence and responsibility*. The first-year students brought up how they developed confidence during the placement when they were entrusted with responsibility to work independently. They were pleased when receiving praise from patients and when they managed to perform a clinical activity on their own without help (“*(My) first time taking a patient’s blood sugar test*. *The pt said that you have done this many times*, *you did it so well*. *So good to hear*. *It went magnificently and the nurse’s assistant was next to me but did not intervene*. *Superfun*!*”*”). Similarly, the final-year students reported that they had developed and were now able to work more independently than in earlier placements. They described how they during rounds had become more comfortable with discussing patients and planning their care with physicians (“…*I round independently now and I am 100% involved*, *today I feel competent to question ordinations and discuss the patients’ care with physicians*”).

Afterwards, a final-year student described how she had been entrusted with responsibility for an entire unit/department and how this made her grow into the role of being a nurse. Another described how a nurse gave her keys and a telephone and let her work independently. She was allowed to plan, organize, and distribute the work and this made her feel that she grew and took part in something meaningful that contributed to her learning.

Another aspect brought up by the students was *ability based on experience and training*. The first-year students often commented that having experience from before and performing a procedure several times enabled them to perform an activity, e.g., catheterization (“…*Second time I’ve done it*. *Quite alright and I’m beginning to feel that I know more than I think*.”). Similarly, the final-year students expressed how previous training enabled them to perform activities, e.g., difficult conversations (“…*I’ve had a lot of use of the dialogue training we got in school*.”).

Afterwards, a first-year student commented that their previous experience from elderly care helped them feel safe (“*I felt that since I had experience from elderly care*, *both home care and [nursing] homes*… *that I was secure from the beginning*”).

During the placements, the first-year students described how they developed competence when *observing others and being supervised*. The students reported that they first watched others perform an activity and then tried on their own and that their supervisors related clinical activities to underlying theory, gave advice and feedback and how this helped them (“*The nurse first explained and recapitulated vaccination theory and then I got to vaccinate a man*. *Went well and she was present and gave tips and feedback*”). One student described how she managed to independently wash, dry, treat, and redress a wound of a patient and to fit a heel protector through observing and receiving guidance from a nurse.

Moreover, during the placements the last-year students commented on their *learning through leading*, *supporting*, *and supervising others* which was something not mentioned by the first-year students. They described talking to and supporting colleagues who did not feel well or were stressed (“*Interesting and a learning experience to support and talk with them*”). They also described how they felt that they learned from supervising (“*Supervised a first-year student when taking a blood sample*. *I learned a lot*!”) and leading others (”…*it has been a learning experience to lead the caring team at this home*…”).

### Reflects critically on competence

The fourth theme encompasses critical reflections about their own competence and of others in relation to what is needed and required at the clinical placement.

During the placements both student groups expressed views which *questioned the competence and behaviors of others*. The students would question the work of nurse’s assistants, nurses, supervisors and expressed that they felt competent enough to question the ordinations of physicians. One first-year student commented that a nurse’s assistant was not competent in taking blood sugar tests (“…*I would have done it better*”). A last-year student objected to her supervisor’s characterization of a patient as “impossible” to interact with and instead emphasized that the patient had integrity and autonomy (“…*the way the supervisor treats* [patients] *at this nursing home is beneath contempt*”). Another final-year student commented that the nurses were not up to date regarding current research. A recurring issue concerned glove wearing routines. When taking blood samples from patients with MRSA and ESBL the students wanted to wear gloves (“*according to guidelines*”) but they described how they were recommended by the supervisors not to, in order to more easily locate blood vessels.

Similar issues were brought up by the first-year students afterwards who would describe that it was confusing when they noted that the staff were using different routines and how this resulted in confusion about which practices that were recommended (“*When everyone does it differently it becomes confusing*”). They described that nurses inserted catheters differently than they had been taught (“…*in school we learned that sterile forceps should be used*…”) and that they were told that it was sufficient to use clean gloves as they were not in a hospital. A first-year student suggested that the personnel may not be aware of how condescending they were towards patients and argued that they needed to take an ethics course.

Afterwards, students expressed *concerns about their own competence*. The first-year students expressed that a challenge had been to understand everything that was new. Doing something the very first time, not knowing enough, or having the right experience—“being a blank slate”—was challenging. These students were overwhelmed (“*Absolutely everything is new*, *I don’t know anything*… *it is very challenging*”) and they had been uncertain about how to act and what to say when a patient had passed away. Another emphasized how she had wanted to make sure that the personnel understood that she did not know anything (“…*I don’t know anything*, *I don’t know anything*, *I have zero experience of health care*…”).

On the other hand, students also expressed *appreciation of knowledge*. One first-year student praised the competence of her supervisor and how important the supervisor had been for her learning (“…*and the nurse was competent*. *She updated us continuously and taught us as much as she could and she was really nice and very orderly*…”). A final-year student expressed how she had felt that her professional competence was appreciated at the placement (“…*they thought it was a relief that we were final-year students because we knew so much*, *that’s what they thought*”).

### Arrangement of the clinical placement

The fifth and final theme concerned the arrangement of the clinical placement including how learning activities, professional roles, and supervision were organized and supported. The final-year students *questioned activities and supervision* at the clinical placements and were critical about how the placements were organized to support their learning (“*it is still not clear to me what they are doing as nurses at this home and how I’m supposed to develop*”). During the placements they reported that their work did not contribute to learning, and that supervisors were not around enough or did not provide enough support and feedback (“*I never get feedback from my supervisor as I’m on my own or with a first-year student most of the time*”).

Afterwards, the final-year students questioned whether they had learned anything during the placement and felt that they were not given opportunities to apply their knowledge (“…*as a nursing student you want to work actively with caring*, *develop plans and interventions*, *evaluate what you’re doing*. *That wasn’t really what they were doing*…”). Both student groups discussed shortcomings of the supervision. Final-year students talked about not being encouraged to participate in clinical tasks and not getting supervision through challenging moments. One student described how her supervisor put on headphones while documenting with the student sitting next to her without inviting her to take part. The student described how this made her feel ill at ease and not knowing what to do. First-year students explained that they would have wanted more supervision especially in the beginning of the placement period when they were uncertain about their role.

Yet another issue brought up by the first-year students afterwards concerned *balancing the roles* of a professional in a clinical environment and that of a student and private person. They expressed concerns about not having enough energy for both working and studying. They were unhappy about having been kept overly occupied with repetitive tasks at the expense of more rewarding learning activities. One student was concerned that she was expected to help the personnel throughout the day doing dishes, serving food and other chores and not being able to fulfil the learning objectives of her study program. Another described how preoccupied she was with thoughts about the patients and had trouble letting go of these (“…*we got connected and it was also a challenge because when I was at home I had difficulties letting go*. *If I was to be home for three days I thought much about—will he be getting his medicine*? *… it was a challenge*”).

In contrast to the more experienced students, the first-year students also brought up the *constructive supervision* that they had received during the placement period. While they experienced having been thrown in at the deep end and being burdened by responsibility, they now in retrospect felt that this had helped them develop professionally. The supervisors entrusted the students with tasks and pushed them to take responsibility for activities which were new to them and which at the time had been experienced as intimidating but in retrospect turned out to be important learning experiences (“… *And it was fun and a learning experience*, *but at the same time scary*. *Now*, *in retrospect*, *I think it was good that she was like that*”).

## Discussion

The importance of clinical education cannot be overstated as it is in the clinical environment that nursing students learn to really understand what it means to be a nurse [20]. Clinical placements outside of traditional hospital settings are predicted to grow in importance [21,22] yet a recent review reported that students fail to see nursing home placements as educationally valuable [7] and another described several challenges faced by students in clinical learning environments [23]. To enhance students’ clinical education, a better understanding is needed. This study contributes by shedding light on nursing students’ experiences during clinical placements not only through interviews afterwards but also by capturing students’ experiences of challenge and competence in the clinical context using an experience-sampling method, combining quantitative assessments with free text comments.

The 575 responses collected in the clinical context showed that the final-year students rated their competence significantly higher than the first-year students. In contrast, the ratings of challenge were significantly higher among the first-year students than among the final-year students.

The reflections made during placements provided valuable accounts of the specifics of the students’ learning activities and the challenges that they encountered. While there was much overlap there were also differences when comparing reflections during and after the placements. Seven categories in two themes were brought up during the placements but not afterwards and, conversely, five categories in three themes were brought up in the interviews but not during the placements (Table 5). Interesting is also that the reflections during and after were somewhat different or even contradictory.

The first theme (*Specific activities are challenging*) concerned the reflections made during the placements focusing on challenges associated with specific clinical activities and were brought up by both first- and final-year students. These activities ranged from taking blood samples and giving injections, documenting and using patient record systems, learning the equipment, interacting with patients who were not perceived as “cooperative”, and talking with patients and their relatives about illness and death. The contrast between the challenging clinical activities during the placements and what the students said afterwards was striking: not a single one of these challenging clinical activities was mentioned in the retrospective interviews. On the contrary, the final-year students expressed how activities during the placements had been under-stimulating and not challenging enough as illustrated by the second theme, *Lack of challenge*. The final-year students reflected on the lack of challenge both during and after the placements and this was clearly something that they wanted to emphasize.

The students’ ratings of clinical activities at the placements showed that the final-year students experienced significantly more boredom than the first-year students. Close to 75% of their responses were categorized as either boredom or apathy implying a lack of challenge (Fig 2). Final-year students’ average ratings of clinical activities were virtually all in the boredom category (Fig 4). In contrast, first-year students experienced significantly more flow and anxiety than the final-year students (Figs 2 & 3). During the placements, the first-year students were often positive and enthusiastic about challenges but many times they also described that they were “scared” “nervous” “insecure” or “not ready” in relation to the clinical activities. In the interviews afterwards, they seemed to view these experiences quite differently. They praised the supervisors for having pushed them to try to carry out activities on their own. While they at the time had not felt ready to take on responsibility for some clinical activities, they were afterwards pleased that they had been pushed beyond their comfort zone. In retrospect they appreciated having gone through these experiences (cf. *Constructive supervision* in the last theme). Unlike the final-year students, the first-year students acknowledged in the interviews that they had been so challenged that they were scared and overwhelmed but they discussed these experiences as valuable opportunities for learning.

Previous research on students’ learning experiences at a clinical training ward has similarly noted discrepancies between experiences reported during clinical practice and how the same students retrospectively describe their clinical practice in questionnaires and interviews [11]: students reported negative experiences including being anxious, nervous, and challenged during their placements, but the same students left such experiences unmentioned afterwards [11]. One explanation may be that it is difficult to remember and describe the details of specific clinical activities and experiences afterwards. What seemed significant at the time may later have been overshadowed by other experiences and may therefore be left unmentioned. The motivation for bringing up such experiences may also have decreased afterwards as they may no longer be as important to the student. Negative experiences may afterwards be reinterpreted as beneficial and strengthening. Problems, challenges, and conflicts which were important at the time may have been resolved and therefore no longer be viewed as relevant. A strength of the experience-sampling method is that it can capture details in context and provide valuable data for researchers and educators which otherwise risk being lost. On the other hand, interviews may elicit overall reflections about the placements which may be easier to generate in hindsight. For instance, the students discussed how they had experienced a lack of structure and requirements at the placements and difficulties combining the roles of being both students and personnel which may be observations that were difficult to do in the midst of an on-going clinical placement.

The third theme, *Learning and developing*, was brought up extensively during the placements and to a lesser degree in the interviews. This theme concerned how students grew confidence when being entrusted with responsibility and working independently and how their own experiences, observing others and supervision impacted on their learning. First-year students would enthusiastically describe how they performed clinical activities on their own after having observed others and without help or presence of the personnel. They emphasized learning through observation and by receiving feedback while the final-year students highlighted how they learned through dialogue with “colleagues” and when supervising younger students. The final-year students appreciated when they had worked independently, participated in rounds and been allowed to be in charge of planning and organizing work. It has been argued that autonomy should not be viewed as a personal trait or an individual quality of the student but rather a social phenomenon which develops in relation to the supervisor, patients and the organization [24]. An implication for clinical education is that it is vital to early on involve the student in ongoing clinical work, not as an outsider, but as one of the team and this, in turn, may lead to meaningful learning (ibid.).

A fourth theme (*Reflects critically on competence*) covered concerns about their own competence and the competence of others. The students reported being overwhelmed by everything that was new and being confused when they noticed that the personnel were not following the guidelines that they had been taught in school, e.g., regarding catheterization or the use of gloves. They praised, but also questioned, the competence of others. Several of the students’ concerns relate to being prepared for work life and thus concern the *theory-practice gap*, the gulf between nursing theory taught in school and clinical practice. Formal education across disciplines has been criticized for not preparing and supporting students in acquiring the needed competences [2,3] and in this context the students found it difficult to follow guidelines and routines that they had learned in school at the placements because other practices were established at the nursing homes or because the students’ approaches were questioned. Both groups of students described difficulties applying evidence-based practices at the nursing homes which led to confusion or questioning the competence and behaviors of the personnel.

Especially the final-year students expressed that the placements should allow students to take on tasks more independently. Their questioning of the existing practices at the placements revealed that they were not content with being passive apprentices at the clinical placements; rather than simply observing and mimicking the behaviors of the employed personnel they expected to be allowed to assume responsibility for their work and to make use their training in ways that may diverge from habits and routines at the placement.

The final theme (*Arrangement of the clinical placement*) concerned critical reflections about the learning activities, supervision and other issues related to how the placements were designed and organized. These included, e.g., remarks about how they felt that their activities at the placements did not support learning or lack of feedback and involvement on the part of the supervisors. The relation to the supervisors has been found to have the greatest impact on how nursing students experience the clinical learning environment in nursing homes and therefore supportive supervision should be ensured didactically as well as organizationally [6]. First-year students commented on the difficulties of combining the conflicting roles of being a student with the aim to learn and develop and the role of working as a nurse. Similarly, Bisholt and colleagues observed that nursing students’ dissatisfaction during clinical placements was due to difficulties with regard to achieving their learning objectives [25].

As seen in the examples above, the final-year students were overall more critical about the level of challenge and the supervision compared to the first-year students. The first-year students on the other hand were positive regarding the learning activities even when they were not challenging. They also expressed more concerns regarding their own competence. A multitude of their reflections expressed delight and pride when they realized that they managed to perform clinical activities independently (“…*I did it on my own without the nurse present*!”). These reflections as well as the quantitative assessments indicate that the final-year students could have been challenged more. On the other hand, the older students’ more critical remarks may also be an indication of maturity. The capacity to critically reflect on one’s practice and to identify possibilities for improvement is a skill which may require extensive experience to develop [26]. It is conceivable that the older students may have developed skills for critically reflecting about their clinical education and that the younger students overlook aspects of clinical activities and supervision that the older students have developed higher expectations about.

Students cannot be expected to experience flow throughout a five-week clinical placement. However, the results raise concerns when almost all the final-year students’ activities are associated with boredom and apathy. From an educational perspective, the placements should challenge and stimulate learning regardless of which semester the student has reached. The lack of professional challenges in nursing homes has been suggested as an explanation to why students consider clinical placements in nursing homes less meaningful than in hospitals [25,27]. An appropriate blend of challenge and support was in one study identified as a key area determining the quality of nursing students’ placement experiences [28]: challenging students can be opportunities for students to discover that they are capable of more than they believe but too much challenge without appropriate support may lead to that students doubt their abilities.

Flow theory offers a model for conceptualizing an appropriate balance between challenges and competence in learning. The added experience and skills of the final-year students means that they need to face other challenges than first-year students to maintain the experienced levels of challenge. These students could for instance be assigned other, more challenging activities or be entrusted with more independence and responsibility in the clinical context. Figs 3 and 4 shows how specific activities were assessed, on an average, by the participants of this study. As has been discussed, retrospective data-collection may not capture such findings and at least not on the same level of detail and accuracy. Consequently, educators and others striving for an appropriate balance between challenge and competence cannot solely rely on what students’ express after their clinical placements.

### Methodological considerations and limitations

This study has explored competence and challenge from different perspectives—both quantitative and qualitative data were collected, during and after clinical placements, and from students on different levels of nursing programmes. A strength is having been able to collect data in context and over time at 21 different nursing homes. The EMA approach relies on intense data collection related to many different experiences of different activities over time which may lessen biases affecting self-reports collected at only one point of time. The data collection was also done close in time to the investigated activities avoiding various forms of memory, recall, hindsight, and fading-affect biases. Patterns uncovered in this study may be transferable to other contexts while specific experiences about clinical placements are likely to vary between individuals and settings. The ratings, responses and reflections are first-person reports and open for the risks associated with self-reports in general. This is a methodological challenge in much of educational research and participants may, e.g., be overconfident in rating their competence levels while underrating challenges. The data collection itself may affect the participants but also be a desired educational side-effect [29]. The experiences of students who chose not to participate could have added additional insights. Repeatedly answering EMA notifications is known to place a considerable burden on participants and study dropout rates are generally high [30]. In a literature review of 461 papers on the use of ESM, van Berkel et al found that the number of participants varied drastically but indicated a median number of 19 participants which can be compared to the 26 participants of this study. They also found a high variation regarding study duration with a median study duration of 14 days and thus equivalent to the duration in this study. Missing responses to planned queries is a methodological concern. The concern for response rates is however dependent on the studied phenomena [30]: in studies investigating long-term conditions, such as dietary patterns, participants with low response rates are of little value whereas studies focusing on short-context situations—such as the clinical activities of this study—can benefit also from participants with small sets of collected data points. In this study there is nevertheless a risk that certain kinds of events, e.g., especially stressful clinical activities, were not included in the EMA assessments which thereby biases the analysis. Moreover, while we made efforts in explaining to the participants that we had a broad interest in all kinds of clinical activities, there is always risk that different participants understand such activities in different ways. Some participants may, e.g., not consider certain types of clinical activities as worth mentioning while others do. In addition, EMA and post-placement interviews are fundamentally different as methods which affects how questions can be asked: EMA studies are oriented towards repeatedly assessing the respondent’s current experience and activity while interviews, for practical reasons, tend to retrospectively generalize over time. To be meaningful, questions need, to some degree, be adapted to the respective method. Such adjustments may affect how questions are interpreted and in turn have effects on findings. Furthermore, while the student groups were within the same educational program and in the same clinical contexts, differences in, e.g., learning objectives may influence the students’ activities and experiences.

The flow analyses were based on the balance between perceived challenges and competence and including aspects such as goals, progress, and feedback could enrichen the analysis. Our analysis has not discriminated between responses made in the beginning, middle, and end of a placements which could potentially have generated further insights. Another limitation of this study is that it relies on the perspective of students. Supervisors and co-workers may have other experiences which could add to the findings of this study.

## Conclusions

Analyses of the data collected about students’ experiences during and after clinical placements showed overlap, but also revealed striking differences. During the placements, numerous clinical activities were reported as challenging but not a single one of these challenges was brought up in the retrospective interviews. Collecting data during the placements, in context, may thus capture a wider range of and more detailed data about clinical activities with less risk for various forms of bias. The momentary assessments can potentially shed light on clinical experiences which for some reason are left out or not remembered afterwards.

On the other hand, a retrospective interview may be an opportunity for reflection which may not present itself in a busy clinical context. Not only did the participants’ retrospective generalizations confirm the main trend observed during the placements that the final-year students had been under-stimulated, the interviews also showed how the participants reinterpreted certain past events. For instance, the first-year students explained that they had been scared and overwhelmed during the placements but that they in hindsight viewed the clinical activities as valuable learning experiences. They appreciated having been pushed to participate although they had not felt ready at the time.

Neither methodological approach provides the full picture. Both have strengths and weaknesses and their different formats may invite different kinds of responses. The retrospective generalizations are interesting as they are likely to give an account which mirrors the students’ lasting experiences of the placements. However, educators, educational designers, and others with an interest in how to best support students’ learning, may especially benefit from students’ contextual assessments instead of only relying on their retrospective generalizations. A strength of a momentary assessment approach is its ecological validity as it can provide the detailed, contextualized data that retrospective methods may overlook.

## Supporting information

S1 Table Estimated means and 95% confidence intervals of students’ ratings of competence and challenge during different weeks of the clinical placements. (DOCX)

S2 Table Competence-challenge associations among first-year students. Means per activity and frequencies.
(DOCX)

S3 Table Competence-challenge associations among final-year students. Means per activity and frequencies.
(DOCX)

S1 Text Excerpts from the free text reflections submitted during clinical placements sorted by theme and category. (PDF)

S2 Text Excerpts from the retrospective interviews sorted by theme and category. (PDF)

## References

[1] Levett-Jones T, Bourgeois S. The Clinical Placement: An Essential Guide for Nursing Students. 2nd ed. Churchill Livingstone Australia: Elsevier; 2011.

[2] Karlgren K, Paavola S, Ligorio MB. Introduction: what are knowledge work practices in education? How can we study and promote them? Research Papers in Education. 2020;35(1):1–7.

[3] Huston C, Phillips B, Jeffries P, Todero C, Rich J, Knecht P, et al. The academic-practice gap: Strategies for an enduring problem. Nurs Forum. 2018;53(1):27–34. doi: 10.1111/nuf.12216 28815609

[4] Killam LA, Mossey S, Montgomery P, Timmermans KE. First year nursing students’ viewpoints about compromised clinical safety. Nurse Educ Today. 2013;33(5):475–80. doi: 10.1016/j.nedt.2012.05.010 22658213

[5] Killam LA, Heerschap C. Challenges to student learning in the clinical setting: A qualitative descriptive study. Nurse Educ Today. 2013;33(6):684–91. doi: 10.1016/j.nedt.2012.10.008 23141689

[6] Carlson E, Idvall E. Nursing students’ experiences of the clinical learning environment in nursing homes: A questionnaire study using the CLES+ T evaluation scale. Nurse Educ Today. 2014;34(7):1130–4. doi: 10.1016/j.nedt.2014.01.009 24529997

[7] Cooke J, Greenway K, Schutz S. Learning from nursing students’ experiences and perceptions of their clinical placements in nursing homes: An integrative literature review. Nurse Educ Today. 2021;100:104857. doi: 10.1016/j.nedt.2021.104857 33714854

[8] Shen J, Xiao LD. Factors affecting nursing students’ intention to work with older people in China. Nurse Educ Today. 2012;32(3):219–23. doi: 10.1016/j.nedt.2011.03.016 21543142

[9] Reis HT, Gable SL, Maniaci MR. Methods for studying everyday experience in its natural context. Handbook of research methods in social and personality psychology. 2014;2:373–403.

[10] Barrett LF, Barrett DJ. An Introduction to Computerized Experience Sampling in Psychology. Social Science Computer Review. 2001;19(2):175–85.

[11] Lachmann H, Ponzer S, Johansson U-B, Karlgren K. Introducing and adapting a novel method for investigating learning experiences in clinical learning environments. Informatics for Health and Social Care. 2012;37(3):125–40. doi: 10.3109/17538157.2012.678449 22713123

[12] Muukkonen H, Inkinen M, Kosonen K, Hakkarainen K, Vesikivi P, Lachmann H, et al. Research on knowledge practices with the Contextual Activity Sampling System. The 9th international conference on computer supported collaborative learning (CSCL); 2009 June 8–13; Rhodes, Greece: ISLS.

[13] Lachmann H, Ponzer S, Johansson U-B, Benson L, Karlgren K. Capturing students’ learning experiences and academic emotions at an interprofessional training ward. Journal of Interprofessional Care. 2013;27(2):137–45. doi: 10.3109/13561820.2012.724124 23043548

[14] Muukkonen H, Lakkala M, Lahti-Nuuttila P, Ilomäki L, Karlgren K, Toom A. Assessing the development of collaborative knowledge work competence: Scales for higher education course contexts. Scandinavian Journal of Educational Research. 2020;64(7):1071–89.

[15] Nakamura J, Csikszentmihalyi M. Flow theory and research. Handbook of positive psychology. 2009:195–206.

[16] Fullagar CJ, Knight PA, Sovern HS. Challenge/skill balance, flow, and performance anxiety. Applied Psychology. 2013;62(2):236–59.

[17] Csikszentmihalyi M, LeFevre J. Optimal experience in work and leisure. J Pers Soc Psychol. 1989;56(5):815. doi: 10.1037//0022-3514.56.5.815 2724069

[18] Holm P, Karlgren K. Cognitive Science on Trial. Proceedings of the 19th Information systems Research seminar In Scandinavia (IRIS); 1996 10–13 August; Lökeberg, Sweden: Gothenburg Studies in Informatics, Report 8.

[19] Paavola S, Hakkarainen K. The Knowledge Creation Metaphor—An Emergent Epistemological Approach to Learning. Science and Education. 2005;14(6):535–57.

[20] Ramani S, Leinster S. AMEE Guide no. 34: teaching in the clinical environment. Med Teach. 2008;30(4):347–64. doi: 10.1080/01421590802061613 18569655

[21] Bjørk IT, Berntsen K, Brynildsen G, Hestetun M. Nursing students’ perceptions of their clinical learning environment in placements outside traditional hospital settings. J Clin Nurs. 2014;23(19–20):2958–67. doi: 10.1111/jocn.12532 24460862 PMC4263152

[22] Laugaland K, Kaldestad K, Espeland E, McCormack B, Akerjordet K, Aase I. Nursing students’ experience with clinical placement in nursing homes: a focus group study. BMC Nurs. 2021;20:1–13. 34488739 10.1186/s12912-021-00690-4 PMC8419895

[23] Panda S, Dash M, John J, Rath K, Debata A, Swain D, et al. Challenges faced by student nurses and midwives in clinical learning environment—A systematic review and meta-synthesis. Nurse Educ Today. 2021;101:104875. doi: 10.1016/j.nedt.2021.104875 33774528

[24] Fredholm A, Savin-Baden M, Henningsohn L, Silén C. Autonomy as both challenge and development in clinical education. Learning, Culture and Social Interaction. 2015;5:20–7.

[25] Bisholt B, Ohlsson U, Engström AK, Johansson AS, Gustafsson M. Nursing students’ assessment of the learning environment in different clinical settings. Nurse Educ Pract. 2014;14(3):304–10. doi: 10.1016/j.nepr.2013.11.005 24355802

[26] Karlgren K, Larsson F, Dahlström A. Eye-opening facilitator behaviours: an Interaction Analysis of facilitator behaviours that advance debriefings. BMJ Simulation and Technology Enhanced Learning. 2020;6(4):220–8. doi: 10.1136/bmjstel-2018-000374 32832101 PMC7410112

[27] Skaalvik MW, Normann HK, Henriksen N. Clinical learning environment and supervision: experiences of Norwegian nursing students—a questionnaire survey. J Clin Nurs. 2011;20(15–16):2294–304. doi: 10.1111/j.1365-2702.2011.03727.x 21752120

[28] Levett-Jones T, Lathlean J, Higgins I, McMillan M. Development and psychometric testing of the Belongingness Scale—Clinical Placement Experience: An international comparative study. Collegian. 2009;16:153–62. doi: 10.1016/j.colegn.2009.04.004 19831149

[29] Lachmann H, Fossum B, Johansson U-B, Karlgren K, Ponzer S. Promoting reflection by using contextual activity sampling: a study on students’ interprofessional learning. Journal of Interprofessional Care. 2014;28(5):7. 10.3109/13561820.2014.907777 24754545

[30] van Berkel N, Ferreira D, Kostakos V. The experience sampling method on mobile devices. ACM Computing Surveys (CSUR). 2017;50(6):1–40.

