# Supplementary material for: ‘Experiencing one thing and saying another’–Ecological Momentary Assessment (EMA) of nursing students’ competence and challenge during clinical placements compared with retrospective interviews
Source: PLoS One. 2024 May 22;19(5):e0302866. doi: 10.1371/journal.pone.0302866 (PMC11111015; doi:10.1371/journal.pone.0302866)
Supplement: S1 Table — (DOCX) [file pone.0302866.s001.docx]

**S1 Table**. Estimated means and 95% confidence intervals of students’ ratings of competence and challenge during different weeks of the clinical placements.

|  | Week 1 | Week 2 | Week 3 | Week 4 | Week 5 |
| --- | --- | --- | --- | --- | --- |
| Competence, first year | 57 (44 – 71) | 64 (53 – 75) | 66 (55 – 78) | 73 (59 – 87) | 76 (64 – 88) |
| Competence, final year | 67 (54 – 79) | 64 (55 – 74) | 82 (66 – 97) | 79 (67 – 91) | 81 (70 – 92) |
| Challenge, first year | 40 (26 – 53) | 46 (35 – 57) | 50 (38 – 61) | 45 (31 – 59) | 40 (29 – 51) |
| Challenge, final year | 29 (16 – 42) | 37 (27 – 47) | 34 (18 – 50) | 25 (12 – 38) | 24 (12 – 35) |
