# Supplementary material for: ‘Experiencing one thing and saying another’–Ecological Momentary Assessment (EMA) of nursing students’ competence and challenge during clinical placements compared with retrospective interviews
Source: PLoS One. 2024 May 22;19(5):e0302866. doi: 10.1371/journal.pone.0302866 (PMC11111015; doi:10.1371/journal.pone.0302866)
Supplement: S2 Table — Means per activity and frequencies. (DOCX) [file pone.0302866.s002.docx]

**S2 Table**. Competence-challenge associations among first-year students. Means per activity and frequencies.

|  |  | | |
| --- | --- | --- | --- |
| Activity | **Competence** | **Challenge** | **Frequency** |
| Administering medicine | 64 | 43 | 22 |
| Blood test | 61 | 52 | 20 |
| Brushing teeth | 64 | 46 | 7 |
| Cleaning and making bed | 85 | 12 | 7 |
| Dialogue | 66 | 39 | 33 |
| Dressing | 89 | 34 | 4 |
| Eating assistance | 74 | 34 | 21 |
| Giving a bed bath | 48 | 55 | 5 |
| Handling catheterization | 56 | 65 | 10 |
| Health maintenance | 82 | 23 | 4 |
| Injection | 57 | 64 | 18 |
| Inserting urinary catheter | 38 | 74 | 4 |
| Mobilizing patient | 69 | 47 | 29 |
| Preparing medicines | 73 | 43 | 25 |
| Rehabilitation | 78 | 72 | 2 |
| Round | 54 | 43 | 8 |
| Showering/washing patient | 73 | 39 | 20 |
| Taking vital signs | 66 | 57 | 7 |
| Toileting assistance | 65 | 43 | 24 |
| Working with patient record | 59 | 52 | 39 |
| Wound dressing | 64 | 58 | 16 |
