# Supplementary material for: ‘Experiencing one thing and saying another’–Ecological Momentary Assessment (EMA) of nursing students’ competence and challenge during clinical placements compared with retrospective interviews
Source: PLoS One. 2024 May 22;19(5):e0302866. doi: 10.1371/journal.pone.0302866 (PMC11111015; doi:10.1371/journal.pone.0302866)
Supplement: S3 Table — Means per activity and frequencies. (DOCX) [file pone.0302866.s003.docx]

**S3 Table**. Competence-challenge associations among final-year students. Means per activity and frequencies.

|  |  | | |  |
| --- | --- | --- | --- | --- |
| Activity | **Competence** | **Challenge** | **Frequency** | |
| Administering medicine | 66 | 35 | 12 | |
| Blood or urine sampling | 93 | 26 | 10 | |
| Dialogue with patient/relative | 77 | 31 | 22 | |
| Following-up drug effects | 79 | 26 | 8 | |
| Help with personal hygiene | 34 | 39 | 2 | |
| Injection | 80 | 29 | 14 | |
| Inserting or managing urinary catheter | 65 | 31 | 10 | |
| Interacting with health care personnel | 72 | 35 | 23 | |
| Leading/planning/organizing care | 71 | 41 | 23 | |
| Managing meal | 50 | 14 | 2 | |
| Mobilizing patient | 53 | 14 | 6 | |
| Preparing medicines | 89 | 8 | 23 | |
| Round | 54 | 33 | 11 | |
| Taking vital signs | 91 | 40 | 17 | |
| Teaching | 59 | 51 | 13 | |
| Working with patient record | 66 | 25 | 25 | |
| Wound dressing | 51 | 43 | 14 | |
