# Supplementary material for: ‘Experiencing one thing and saying another’–Ecological Momentary Assessment (EMA) of nursing students’ competence and challenge during clinical placements compared with retrospective interviews
Source: PLoS One. 2024 May 22;19(5):e0302866. doi: 10.1371/journal.pone.0302866 (PMC11111015; doi:10.1371/journal.pone.0302866)
Supplement: S1 Text — (PDF) [file pone.0302866.s004.pdf]

Excerpts from the free text reflections submitted during clinical placements sorted by theme and category

The excerpts have been translated into English and are sorted by theme and category as presented in Table 5 of the Results/Findings. Blue excerpts are from the first-year students' reflections and red excerpts from the final-year students' reflections.

Specific activities are challenging

Clinical procedures

"Patient whose pressure was difficult to measure. But together with another student we managed to get it done." (First-year student)

"Injected Innohep subcutaneously in the abdomen. It went well but was a bit difficult to get through the skin the first time." (First-year student)

"Special case [of replacing an indwelling urinary catheter] as the patient has fistula passages making it difficult to find your way. The right method is difficult to apply as the catheter ended up in the wrong opening. Then the nurse left it in so that we could find the urethra more easily. Worked well on the second try." (First-year student)

"Difficult to give subcutaneous injections to somebody without any body fat at all. I find it scary to give subcutaneous injections to someone who is extremely thin." (Final-year student)

"Difficult to pass out [medication] properly and efficiently with old drug lists" (Final-year student)

"I often follow up on drugs that ease pain. Some pains are difficult to relieve, and it becomes difficult to find the right medicine for them. Moreover, I assess the effect after having administered Furix. I don't feel very knowledgeable about the medical side." (Final-year student)

"Blood sampling with difficulties to find vessels. Patient with MRSA and ESBL. Therefore, I want to wear gloves." (Final-year student)

Documentation

"Been reading in a patient's record. Interesting but many difficult words and abbreviations" (First-year student)

"Read anamnesis/journal. Difficult to learn all of the new language." (First-year student)

"Difficult to document wounds in the patient record system. There are no "predefined" words to choose between, only free text [is allowed]. Feels challenging" (Final-year student)

Interaction with the patient

"Dialogue about loneliness and death, difficult to know what to answer... a lot of worry and anxiety among those living in the [nursing home]" (First-year student)

"Difficult to brush when the resident doesn't want to open his/her mouth and bites the toothbrush"

"The resident refuses to take the medicine, I try again and again. Difficult situation." (Final-year student)

"Difficult ethically as it is a patient with dementia and communication is limited." (Final-year student)

Interaction with relatives

"Conversing with relatives whose mother is in the end stage of palliative care. Difficult, when relatives ask why their mother is dying, etc. You have to think through your answers carefully and explain that this person is on a natural path towards death due to the disease." (Final-year student)

"Difficult conversation with a relative who expressed painful feelings about an incident with a parent. Hard to know what to say." (Final-year student)

Learning the equipment

"Helped patients get out of bed and on to a wheelchair, walking frames or rollator. Difficult, however, to know exactly how the wheelchair/walking frame/rollator works, with the brake, to raise/lower [the height] etc." (First-year student)

"I have never seen such an air mattress. Didn't know how to remove it from the bed, open the lid to let the air out." (First-year student)

Lack of challenge

Tasks are repetitive or under-stimulating

"Day 3 at the clinical placement. I am feeling very under-stimulated at this nursing home, most have ApoDos, [pre-dosed and prepackaged medicine], some insulin. Control of blood pressure and pulse, weight and patch change once a week. A lot of time is spent by the staff talking about each other's and the organization's shortcomings. I don't have access to a login to read medical records or to document independently." (Final-year student)

"I have taken vital parameters so much during my studies and work that it is not stimulating for me anymore" (Final-year student)

Tasks are easy but important

"I administered an ApoDos to the patient. Not so difficult but important! Patient grateful!" (First-year student)

"I handed out dosettes [pill organizers] to the residents, not especially challenging but an important task in the profession" (First-year student)

Learning and developing

Confidence through independence and responsibility

"(My) first time taking a patient's blood sugar test. The pt said that you have done this many times, you did it so well. So good to hear. It went magnificently and the nurse's assistant was next to me but did not intervene. Superfun!" (First-year student)

"Now I feel comfortable taking patients' blood pressures. I did it on my own without the nurse present." (First-year student)

"Took care of mobilizing a resident on my own. I have seen before how the staff had done it and now I did it myself without anyone else in the room. Went very well." (First-year student)

"In comparison to what it was like in the second semester, I round independently now and I am 100% involved, today I feel competent to question ordinations and discuss the patients' care with physicians." (Final-year student)

"I have done ward rounds of the patients I have had for 3 weeks. I feel safe in telling what the doctor should know. For example, [I] told the doctor about a blood sugar curve that was completely out of balance which led to that the doctor then changed the insulin dose. I told [him/her] that this imbalance occurred in connection with the introduction of cortisone." (Final-year student)

"Ward round on my own with the doctor. Experiencing this last week as instructive as I think I have a good grasp of the 11 patients we have at the ward. Very interesting to discuss and collaborate on my own with doctors." (Final-year student)

Ability based on experience and training

"Drained a patient of urine with a clean intermittent catheter. Second time I've done it. Quite alright and I'm beginning to feel that I know more than I think" (First-year student)

"Given 34 units of insulin in the abdomen to a resident. Everything went well, I have performed this task MANY times at my regular workplace so there were no oddities." (First-year student)

"Conversation with a next of kin whose relative has been exposed to serious trauma. I've had a lot of use of the dialogue training we got in school." (Final-year student)

"[I] work independently taking samples ... Good to practice on old [patients] who are difficult to inject, I guess ... never had difficulties with it and have done it a lot when I worked as a nurse's assistant." (Final-year student)

Observing others and being supervised

"The nurse first explained and recapitulated vaccination theory and then I got to vaccinate a man. Went well and she was present and gave tips and feedback afterwards." (First-year student)

"I redressed a heel wound on a man. I washed with soap and water, dried, applied remedy and bandages, dressed, and fitted the heel protector in place. Everything went well, I performed the part independently but under the observation and guidance of my nurse" (First-year student)

"I was just observing. Feels like I can handle it on my own next time." (First-year student)

"I learned by watching when the nurse took tests and then I've tried on the head of the clinic, and now I got to try on a patient. Felt good afterwards and the patient was satisfied!" (First-year student)

Learning through leading, supporting, and supervising others

"Meeting with the nurse's assistants. They have talked about how bad they feel at work. It has been very interesting to learn how to talk to colleagues who don't feel well and are stressed. Interesting and a learning experience to support and talk with them". (Final-year student)

"Supervised student from the second semester on blood sampling. Very much of a learning experience!" (Final-year student)

"I feel that I learn from leading the nursing team at this facility. It has been instructive to collaborate so closely…" (Final-year student)

Reflects critically on competence

Questions the competence & behaviors of others

"Participated in taking blood sugar, the nurse's assistant was not at all competent. Felt like I would have done it better. The student who is at the same nursing home (she is a nurse's assistant) had to take over and complete the test so that the patient would not have to experience more suffering." (First-year student)

"I was warned by the supervisor that the patient is impossible. That she fights and that it is impossible to carry out steps such as redressing her sacrum wound or giving insulin. After meeting the patient continuously and performing the steps myself, I have absolutely not noticed this! The patient has great integrity and self-determination that needs to be met, promoted and respected! The way the supervisor treats [patients] at this nursing home is beneath contempt!" (Final-year student)

"However, poor feedback and communication in general at the nursing home. Lack of knowledge regarding new research, etc. from the nurses as well." (Final-year student)

"The patient has ESBL and MRSA so I wear gloves and an apron when helping the patient to eat at the bedside. However, it feels a bit "offensive" with gloves etc. when feeding. The nurse's assistants say it is offensive, but the nurse thinks it's great. According to guidelines, gloves and an apron must be used when working close to patients". (Final-year student)

"…Therefore, I want to wear gloves. The supervisor recommends feeling and looking for vessels without gloves to find vessels." (Final-year student)

Arrangement of the clinical practice

Questions activities and supervision

"Nice reception, but it is still not clear to me what they are doing as nurses at this home and how I'm supposed to develop." (Final-year student)

“Supervisors never take part and I experience poor support and feedback. [The only] feedback after having handed out medication, is, right away, that you should run off and do something else.” (Final-year student)

“I never get feedback from my supervisor because I work most of the time on my own or with a second-semester student. Taking vital signs is one of the most boring tasks and I am not learning anything new from it.” (Final-year student)

“Flushed an indwelling urinary catheter. I feel safe and satisfied after performing a procedure on a patient. However, you don’t get any response and you don’t get to discuss properly with supervisors either.” (Final-year student)
