# Supplementary material for: ‘Experiencing one thing and saying another’–Ecological Momentary Assessment (EMA) of nursing students’ competence and challenge during clinical placements compared with retrospective interviews
Source: PLoS One. 2024 May 22;19(5):e0302866. doi: 10.1371/journal.pone.0302866 (PMC11111015; doi:10.1371/journal.pone.0302866)
Supplement: S2 Text — (PDF) [file pone.0302866.s005.pdf]

Excerpts from the retrospective interviews sorted by theme and category

The excerpts have been translated into English and are sorted by theme and category as presented in Table 5 of the Results/Findings. Blue excerpts are from interviews with first-year students and red excerpts from interviews with final-year students.

Specific activities are challenging

(None)

Lack of challenge

Tasks are repetitive or under-stimulating

”Now after these weeks I feel the same as I experienced all the time: Every day, I felt: What am I doing here? What is this good for? Why have they chosen this? Many times, we did not learn anything new and I just twiddled my thumbs. I found that a lot of time was spent just talking about private things, and most people talk about how hard it is with the elderly and their relatives.” (Final-year student)

“It felt like [the clinical placement] was very much of the same thing all the time. Many activities were very similar so not that much happened. Feels as if time could have been used in a better way. Overall, it was not particularly challenging, and that is why I sort of feel that time could have been used in a better way.” (Final-year student)

Lack of structure and requirements

”I still felt that this supervisor was not really interested in having a student, in that she did not supervise as she should. She didn't teach. She did not reflect in the same way that a supervisor should. I didn't even feel like a colleague to her but her tail or appendage, and that feeling isn’t nice either. So unfortunately, my fears turned out a bit like I had imagined.” (Final-year student)

”Once I was there, the arrangement felt messy for me as a student. There was no structure. It felt as if I had to create a structure myself to make [the clinical placement] make sense. I got a supervisor who accepted me, it was very nice and so on but [he/she] never made a plan for the day. Many times, it felt like I had to sit there and twiddle my fingers and didn’t really know what to do because I hadn’t gotten an introduction, contact with the patients, I couldn't just get up, walk in, and do things independently.” (Final-year student)

Tasks are easy but important

”After the clinical placement, I thought it through myself. At first I thought that ’the first week was unnecessary for me because I [already] have some healthcare experience. It is unnecessary for me to repeat this’, but in retrospect I thought, no, it was rewarding. I got to know the fantastic residents. It was a dementia home. So actually, it was always rewarding if you think about it, but not in the beginning. It was a good workplace for me. …” (First-year student)

Learning and developing

Confidence through independence and responsibility

“I had to take a lot of responsibility. I got to run my own ward. So, it was great fun. … because you grew into the role of a nurse which feels important so close to the end [of the study program]” (Final-year student)

“... I just sat a lot of the time until I got to work with a substitute nurse and then I experienced that her approach was completely different. She gave me keys, a phone, [and let me take] command. She said ’I'm your shadow, I’m behind you but you get to work independently, and this is what the day is like, this is what we are going to do: You plan, organize, distribute the work in a way that feels good for you’. And thereby I was allowed to grow. So Mondays were wonderful because I knew I was going to work with this substitute nurse and I actually felt like I was doing something meaningful and useful and something that I was learning from.” (Final-year student)

Ability based on experience and training

”I felt that since I had experience from elderly care, both home care and [nursing] homes, that I was a bit secure from the beginning” (First-year student)

Reflects critically on competence

Questions the competence & behaviors of others

”Then the assistant nurse showed and I had to try doing the same thing. We had done a lot at the clinical training center, so you had a bit of an idea of that. How to wash and in which direction. What equipment should I wear? It also meant that you had to think ”What am I going to do now? Should I wear an apron or not?”. When everyone does it differently, it becomes confusing: ’How was it?’ then you can go back to The Handbook for Healthcare and check: ”Should it, or should it not?”” (First-year student)

”I think theory and practice must be connected. Sometimes when you come down to reality, it’s not exactly like that … What we learn to do in school - and if we look at how nurses perform the same task - it’s not the same routines. As a student, I think that there is a healthcare law, there is The Handbook for Healthcare that gives us guidelines and recommendations on how to act. It's because we want to be safe. The information found in, for example, The Handbook for Healthcare is based on proven experience, and this is important because the law states that care must be based on evidence-based experience. … I wondered, for example, why nurses don't use sterile forceps when inserting a catheter. I got the answer that ”We use gloves, they are clean, this is not a hospital. That should be enough”. But in school we learned that sterile forceps should be used.” (First-year student)

“I think that the staff, especially assistant nurses, might need a small course in ethics. How to treat people. They say something not intending to sound hurtful, but to others listening it may sound a little degrading. For example, when someone talks over the patient’s head, or when you refer to a patient using the room number. E.g., "Number 206 needs diapers”.” (First-year student)

Concerns about their own competence

”I was like this at the beginning ”I don’t know anything, I don’t know anything, I have zero experience of health care, I don’t know anything” all the time. [I] wanted to assure them that this was really the case, ”You have to listen to me.” …It’s probably more about me accidentally doing something wrong and not having someone with you who is in charge. I never thought that it would lie upon me, that I could get hurt. Rather, I felt that I could trust them and if things went wrong, they would sort it out. But you never know.” (First-year student)

“But I guess I thought that everything is new to me. Absolutely everything is new, I don’t know anything, I don’t know anything. I was like being a blank slate. So I think that everything that I will do, or everything that will happen is new to me. And then I think that it is very challenging, what happened to me was ”I don't know or I am not able”. Because I felt during this period that there is a lot to take in, all the time. There’s a lot. I had to constantly take notes in the logbook, remember everything.” (First-year student)

“I had not worked in healthcare before, I had work with other things. Therefore, it was very challenging for me in the beginning. … Everything was new. It’s hard to do things the first time.” (First-year student)

“… when we got there the first day, I think, a person had passed away a few weeks earlier and they were going to have a memorial service for that person. Then I and the other student who was there were a little unsure of what to do. After all, we didn't know any of the staff, or relatives, or the lady who had passed away. ... It was a bit of a ”what do you do here then?” situation” (First-year student)

Appreciation of knowledge

”After that it was fantastic, and the nurse was competent. She updated us continuously and taught us as much as she could, and she was really nice and very orderly. When I started working with a nurse, it felt great to me” (First-year student)

”I felt that it was open, that we could talk about things, that we were appreciated. That they thought it was a relief that we were final-year students because we knew so much, that's what they thought … I think they found it a relief that we were there because we helped so much, and in the end, it was like we worked there. You could kind of joke about it that "but can't you come here this weekend and help?". So it was fun in that way that you were so... you felt appreciated” (Final-year student)

Arrangement of the clinical practice

Questions activities and supervision

“In the beginning it didn't feel very good. I didn't know what my role was. I've brought it up with my supervisor, but we haven't really gotten an answer. They have only told us that the curriculum will be followed. So in the beginning it was a bit difficult. I was a bit worried that I will be stuck here and not learn what I'd like to learn and according to curriculum. It was not a good start the first week. It was unclear. It's not just my experience, I've asked other students too. ....” (First-year student)

”It's difficult when you come to a business like that, especially a private one. For example, as a nursing student you want to work actively with caring, develop plans and interventions, evaluate what you're doing. That wasn't really what they were doing. And then it becomes very difficult. I'm not there all hours of the day. And if the others are not onboard - and are there as nurses - then it becomes very difficult to get a sense of continuity in what you are doing and also confirmation that you are doing things in a good way.” (Final-year student)

”[The supervisor] would sometimes put on headphones when she was documenting and I was sitting next to her. … I was sitting there. So I really felt ill at ease many times because I really didn't know what to do. Should I go away and find something to do, or what do I do? One day I got up and cleaned the medicine room and sorted all the medicines, organized and so on.” (Final-year student)

”…because I feel that nothing has been good about this practice. And I was three weeks on another clinical placement and I felt like ”God, I've learned a lot”. While elderly care was five weeks. … Yeah, it was like… I didn't think it was for real many times.” (Final-year student)

”Yes, I would have liked more challenge and to be supervised in these challenges. To get to know ”Okay, this is how I think. How is it done in the best possible way? What do you think?”. To get a moment for reflection. If you encounter difficult moments like this later, you're left completely helpless because you've never analyzed, or come close to understanding, what you can do in such a situation, you kind of do not get the picture. So challenges? Absolutely, I would have liked more.” (Final-year student)

Balancing roles

“There was a lack of staff there when we started. It was always like that. My partner, the other student, she started helping with all sorts of things. Packing things, the dishes, serving, and anything possible. What worried me was that I wouldn't have time to observe other important things that my curriculum says to follow. She didn't say anything, she didn't speak up. I had to tell the assistant nurse that "I will get to know and have contact with my patient that I will write about". I took a bit of the hit, I spoke out. I want it to be clear and distinct. If I'm going to help them - the staff - all day, it's going to be hard for me. I will be completely exhausted when I get home because we also have to study at home. We have to do that and look at other course literature. It is important to us. So, I think that was the part I was worried about.” (First-year student)

”Because I like him [a patient] a lot and we got connected and it was also a challenge because when I was at home I had difficulties letting go. If I was to be home for three days, I thought much about - will he be getting his medicine? And it's for his epilepsy anyway. It's no good at all for him if he doesn't take his epilepsy medicine. It was a challenge. So maybe that's how I've defined challenge.” (First-year student)

”I think that I wouldn't have had to shower different residents three times, once would have been enough for me. I didn't have to shower three times. I have a little energy and that energy should be used for rewarding useful things for me that I would like to learn” (First-year student).

Constructive supervision

“I got a nurse who really, I don’t know how to describe it, but she let me do everything. And she thought that even if I was uncertain about any part, she was there in the background. She said it ”I'm here, ask anything". She pushed me a lot. She said she had worked twenty-nine years, I think, as a nurse. She even asked me if I wanted to do things we hadn't tried at the clinical training center, such as venous sampling. And it was perfectly fine when I said I didn’t want to. But she said "Well, consult The Handbook for Healthcare, read, and tomorrow you’ll do the next one". I don’t know, but I thought she supported me a lot.” (First-year student)

”...So she [the supervising nurse] used to push me sometimes: "Now you go in and you take and control this, [name of the student]”. And it was fun and a learning experience, but at the same time scary. Now in retrospect, I think it was good that she was like that.” (First-year student)
